# Suppression of Aflatoxin Biosynthesis in *Aspergillus flavus* by 2-Phenylethanol Is Associated with Stimulated Growth and Decreased Degradation of Branched-Chain Amino Acids

**DOI:** 10.3390/toxins7103887

**Published:** 2015-09-24

**Authors:** Perng-Kuang Chang, Sui Sheng T. Hua, Siov Bouy L. Sarreal, Robert W. Li

**Affiliations:** 1 Southern Regional Research Center, Agricultural Research Service, U. S. Department of Agriculture, New Orleans, LA 70124, USA; 2 Western Regional Research Center, Agricultural Research Service, U. S. Department of Agriculture, Albany, CA 94710, USA; E-Mails: sylvia.hua@ars.usda.gov (S.S.T.H.); siov.sarreal@ars.usda.gov (S.B.L.S.); 3 Animal Genomics and Improvement Laboratory, Agricultural Research Service, U. S. Department of Agriculture, Beltsville, MD 20705, USA; E-Mail: robert.li@ars.usda.gov

**Keywords:** *Aspergillus flavus*, 2-phenylethanol, aflatoxin, gene ontology, metabolic pathway, functional genomics

## Abstract

The saprophytic soil fungus *Aspergillus flavus* infects crops and produces aflatoxin. *Pichia anomala*, which is a biocontrol yeast and produces the major volatile 2-phenylethanol (2-PE), is able to reduce growth of *A. flavus* and aflatoxin production when applied onto pistachio trees. High levels of 2-PE are lethal to *A. flavus* and other fungi. However, at low levels, the underlying mechanism of 2-PE to inhibit aflatoxin production remains unclear. In this study, we characterized the temporal transcriptome response of *A. flavus* to 2-PE at a subinhibitory level (1 µL/mL) using RNA-Seq technology and bioinformatics tools. The treatment during the entire 72 h experimental period resulted in 131 of the total *A. flavus* 13,485 genes to be significantly impacted, of which 82 genes exhibited decreased expression. They included those encoding conidiation proteins and involved in cyclopiazonic acid biosynthesis. All genes in the aflatoxin gene cluster were also significantly decreased during the first 48 h treatment. Gene Ontology (GO) analyses showed that biological processes with GO terms related to catabolism of propionate and branched-chain amino acids (valine, leucine and isoleucine) were significantly enriched in the down-regulated gene group, while those associated with ribosome biogenesis, translation, and biosynthesis of α-amino acids were over-represented among the up-regulated genes. Kyoto Encyclopedia of Genes and Genomes (KEGG) pathway analysis revealed that metabolic pathways negatively impacted among the down-regulated genes parallel to those active at 30 °C, a condition conducive to aflatoxin biosynthesis. In contrast, metabolic pathways positively related to the up-regulated gene group resembled those at 37 °C, which favors rapid fungal growth and is inhibitory to aflatoxin biosynthesis. The results showed that 2-PE at a low level stimulated active growth of *A. flavus* but concomitantly rendered decreased activities in branched-chain amino acid degradation. Since secondary metabolism occurs after active growth has ceased, this growth stimulation resulted in suppression of expression of aflatoxin biosynthesis genes. On the other hand, increased activities in degradation pathways for branched-chain amino acids probably are required for the activation of the aflatoxin pathway by providing building blocks and energy regeneration. Metabolic flux in primary metabolism apparently has an important role in the expression of genes of secondary metabolism.

## 1. Introduction

*Aspergillus flavus*, which is a common plant and an opportunistic human pathogen, can produce the carcinogenic aflatoxin. Contamination of crops such as corn, cotton, peanuts, and tree nuts by aflatoxin poses a great food safety risk especially in developing countries due to ineffective farming practices and the lack of proper storage facilities. Aflatoxin contamination can also result in devastating economic losses because of strict regulations on dissemination of contaminated products [1]. Currently, there are no commercially available fungal cultivars resistant to the infection by *A. flavus*. The only promising intervention method showing measurable extents of control of aflatoxin contamination is to use biological control, which includes applying atoxigenic *A. flavus* strains, such as AF36 [2], K49 [3] or Afla-Guard^®^ [4], to outcompete toxigenic strains in the field or spraying a yeast formulation to pistachio trees to prevent fungal growth [5]. In field tests, these biocontrol approaches have achieved greater than 80 percent reduction in aflatoxin contamination.

*Pichia anomala* WRL-076 is the only biocontrol yeast that has been shown to inhibit growth and aflatoxin production of *A. flavus* [6,7]. Most recently, 2-phenylethanol (2-PE) has been identified as the major volatile compound produced by this yeast [8]. 2-PE is widely found in nature, especially in flower extracts and fragrant essential oils. It has a pleasant floral odor and thus is a common ingredient of perfume. Yeast such as *Candida albicans* [9], *Kluyveromyces marxianus* [10], *Saccharomyces cerevisiae* [11] and *Kloeckera apiculata* [12] also produce 2-PE. This volatile has been demonstrated to have inhibitory properties against *Penicillium italicum*, which causes postharvest citrus decay [12]. The underlying mechanisms of 2-PE inhibition on growth at high concentrations have been reported on bacteria and fungi, which mainly disrupt organelles like mitochondria and nucleus, and synthesis of macromolecules, such as enzymes [13].

A better understanding of the mode of action of 2-PE at low concentrations, a scenario likely to be encountered in field applications of the biocontrol yeast, is critical to the development of an effective biocontrol formulation. At subinhibitory levels to fungal mycelial growth [8], how 2-PE affects aflatoxin biosynthesis is still not well understood. The objective of this study was to use the RNA-Seq approach to determine transcriptomic changes in *A. flavus* treated by a subinhibitory concentration (1.0 µL/mL) of 2-PE and to examine whether changes in the expression of specific genes of certain metabolic pathway had a bearing on inhibition of aflatoxin production. At this low concentration, 2-PE mostly stimulated fungal growth as evidenced by gene ontology (GO) enrichment analyses showing the increased structural constituent of ribosome and an active translation (α-amino acid biosynthesis). The outcomes, along with a decrease in the degradation of branched-chain amino acids, were correlated with the suppression of all aflatoxin pathway gene expression.

## 2. Results

### 2.1. Summary of RNA-Seq Datasets and Statistic Analysis

**Figure 1:**
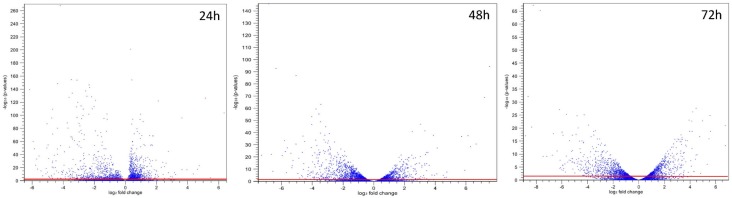
Volcano plots showing original *p*-values *versus* fold change. Each point represents the results of a gene derived from the comparison of three 2-PE treated replicate samples to those untreated samples. The *x*-axis is the log2 transformed fold change in gene expression after the 2-PE treatment. The *y*-axis is the −log10 transformed original *p*-value. A *p*-value of 0.05 is equivalent to the value of 1.30 in the –log10 transformed *y*-axis. Genes above the (red) line are those differentially expressed (*p* < 0.05).

The sum of single-end reads of the three biological replicates obtained from each of the experimental conditions that passed the quality control procedures ranged from 59 to 92 million (Table S1). Of the total 433 million reads, about 66.5% were mapped uniquely to the gene regions of *A. flavus* NRRL3357. Among these reads about 96.9% were located in the exon regions and 3.1% were located in the intron regions. Volcano plots derived from the 24 h, 48 h and 72 h gene expression data showing original *p*-values on the *y*-axis and fold change on the *x*-axis was generated (Figure 1). The overall fold changes at these three time points did not vary greatly, but the *p*-value range changed from E-270 at 24 h to E-67 at 72 h, which indicated a decreasing trend in the significance of differentially expressed gene as cultures aged.

Statistical analyses using the “Exact Test” on the RPKM counts with the total count filter cutoff of 5 and the FDR (False Discovery Rate) correction of *p* < 0.05 were performed to eliminate those false-positive genes that were initially considered positive based on original *p*-values. Table 1 summarizes the corrected numbers of differentially expressed genes obtained at a single or combined time points, which represent different growth periods. The final numbers of genes differentially expressed at these periods reflected the trend observed from the volcano plots (Table S2). Depending on the time point or the period examined, the number of differentially expressed genes based on the corrected p-values decreased to 25%–50% compared to those based on the original *p*-value. Only 131 of the total 13,458 genes in the 2-PE treated cultures were differentially expressed during the entire 72 h period (Table 1). Clustering genes for cyclopiazonic acid biosynthesis (AFLA_139470, AFLA_139480, and AFLA_139490) and genes for conidiation proteins (AFLA_044790, AFLA_044800, and AFLA_083110) were among these significantly down-regulated during the 72 h period.

**Table 1:** Differentially expressed genes in the 2-PE treated *A. flavus* at different growth periods.

Expression ^a^	Time					
	24 h	48 h	72 h	24 & 48 h	48 & 72 h	24 & 48 & 72 h
Decreased	967	959	671	255	291	82
Increased	1200	593	527	163	197	49

a: FDR corrected *p*-value is <0.05.

### 2.2. Treatment by 2-PE Decreased Expression of All Genes in the Aflatoxin Gene Cluster of A. flavus

For a better understanding of the effect of 2-PE on the aflatoxin gene expression by *A. flavus*, all genes in the aflatoxin biosynthesis gene cluster were examined (Table 2). The decrease in gene expression was most prominent at 24 h and ranged from two- to greater than 18-fold. In comparison, the decrease at 48 h and 72 h was within three-fold. *A. flavus* can only produce aflatoxin B_1_ and B_2_. The expression pattern of *norB* and *cypA*, which are involved in the biosynthesis of aflatoxin G_1_ and G_2_, were less correlated with that of other genes responsible for B-type aflatoxin production. Although fold of decreases at 48 h and 72 h were comparable, all values at 72 h after the FDR correction were not significant (FDR corrected *p*-values > 0.05, Table 2). This was found to be caused by increased variance for each gene at 72 h compared to its counterpart at 24 h and 48 h, respectively.

**Table 2:** Fold change in expression of genes in the aflatoxin gene cluster.

Gene ID	Gene Name and Product	24 h	FDR	48 h	FDR	72 h	FDR
AFLA_139140	*aflYa*/*nadA*/NADH oxidase	−5.91 ^a^	1.00	−1.89	0.00	−2.67 ^a^	0.07
AFLA_139150	*aflY*/*hypA*/*hypP*/hypothetical protein	−10.32	0.00	−2.39	0.00	−2.17 ^a^	0.30
AFLA_139160	*aflX*/*ordB*/monooxygenase/oxidase	−7.33	0.00	−1.56	0.00	−2.07 ^a^	0.31
AFLA_139170	*aflW*/*moxY*/monooxygenase	−8.36	0.00	−2.05	0.00	−2.09 ^a^	0.35
AFLA_139180	*aflV*/*cypX*/P450 monooxygenase	−11.59	0.00	−2.12	0.00	−2.03 ^a^	0.40
AFLA_139190	*aflK*/*vbs*/VERB synthase	−5.67	0.00	−2.98	0.00	−2.50 ^a^	0.14
AFLA_139200	*aflQ*/*ordA*/P450 monooxygenase	−10.57 ^a^	0.07	−1.87	0.00	−2.11 ^a^	0.29
AFLA_139210	*aflP*/*omtA*/*omt1*/*O*-methyltransferase A	−14.69	0.00	−2.27	0.00	−2.03 ^a^	0.41
AFLA_139220	*aflO*/*omtB*/*dmtA*/*O*-methyltransferase B	−5.83	0.00	−1.45	0.03	−1.62 ^a^	0.74
AFLA_139230	*aflI*/*avfA*/P450 monooxygenase	−21.13	0.02	−2.28	0.00	−2.11 ^a^	0.21
AFLA_139240	*aflLa*/*hypB*/hypothetical protein	−13.51	0.00	−2.46	0.00	−2.37 ^a^	0.16
AFLA_139250	*aflL*/*verB*/desaturase/P450 monooxygenase	−8.64	0.00	−2.44	0.00	−2.36 ^a^	0.15
AFLA_139260	*aflG*/*avnA*/*ord1*/P450 monooxygenase	−5.09 ^a^	0.19	−1.74	0.00	−2.17 ^a^	0.20
AFLA_139270	*aflNa*/*hypD*/hypothetical protein	−3.32	0.00	−1.09 ^a^	0.34	−1.76 ^a^	0.46
AFLA_139280	*aflN*/*verA*/monooxygenase	−4.29 ^a^	0.11	−1.58	0.00	−2.27 ^a^	0.18
AFLA_139290	*aflMa*/*hypE*/hypothetical protein	−6.94	0.00	−1.41	0.01	−1.74 ^a^	0.56
AFLA_139300	*aflM*/*ver1*/dehydrogenase/ketoreductase	−18.07	0.00	−1.79	0.00	−1.83 ^a^	0.55
AFLA_139310	*aflE*/*norA*/*aad*/*adh2*/NOR reductase	−10.11	0.00	−1.86	0.00	−2.32 ^a^	0.19
AFLA_139320	*aflJ*/*estA*/esterase	−4.95	0.00	−1.42	0.01	−2.15 ^a^	0.29
AFLA_139330	*aflH*/*adhA*/short chain alcohol dehydrogenase	−4.75	0.00	−1.78	0.00	−2.22 ^a^	0.22
AFLA_139340	*aflS*/*aflJ/*pathway regulator	−2.67	0.00	−1.12 ^a^	0.25	−1.10 ^a^	1.00
AFLA_139360	*aflR*/*apa2/afl2*/C6 transcription factor	−2.00	0.02	−1.10 ^a^	0.43	−1.40 ^a^	0.72
AFLA_139370	*aflB*/*fas1*/fatty acid synthase beta subunit	−2.54 ^a^	0.24	−1.79	0.00	−1.58 ^a^	0.52
AFLA_139380	*aflA*/*fas2*/*hexA*/fatty acid synthase alpha subunit	−1.27 ^a^	1.00	−2.13	0.00	−2.29 ^a^	0.09
AFLA_139390	*aflD*/*nor1*/reductase	−3.46	0.00	−2.33	0.00	−2.29 ^a^	0.21
AFLA_139400	*aflCa*/*hypC*/hypothetical protein	−4.53	0.00	−2.60	0.00	−2.66 ^a^	0.08
AFLA_139410	*aflC*/*pksA*/*pksL1*/polyketide synthase	−2.01 ^a^	0.59	−2.32	0.00	−2.57 ^a^	0.12
AFLA_139420	*aflT*/transmembrane protein	−5.12 ^a^	0.62	−1.53	0.00	−1.79 ^a^	0.15
AFLA_139430	*aflU*/*cypA*/P450 monooxygenase	−4.92 ^a^	1.00	−1.27 ^a^	0.59	−1.29 ^a^	1.00
AFLA_139440	*aflF*/*norB*/dehydrogenase	−7.48 ^a^	1.00	−1.18 ^a^	0.71	−1.26 ^a^	1.00

^a^: The FDR > 0.05.

### 2.3. GO Functional Classification of Differentially Expressed Genes

**Figure 2:**
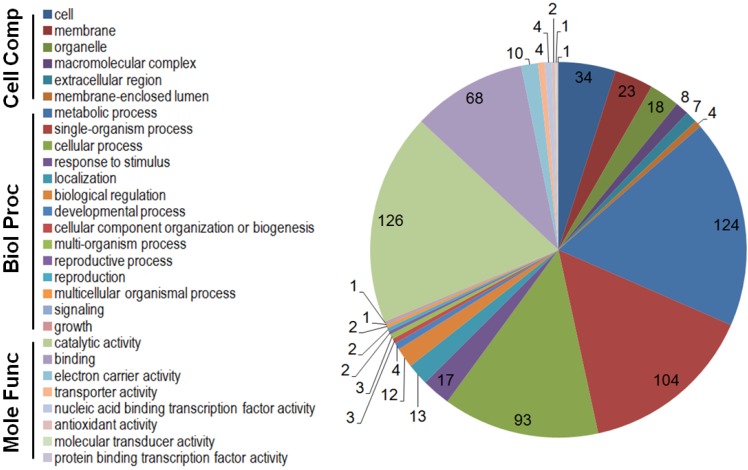
GO categories and GO terms of the 255 significantly down-regulated genes during the first 48 h growth period. The three categories are cellular component, biological process and molecular function. The combined graphic was generated using the GO level 2.

In light of the enlarged variance for the aflatoxin gene expression level at the late stage of growth, we arbitrarily selected 48 h as the cut-off time for evaluating 2-PE treatment on GO functional categories of the differentially expressed genes. The treatment resulted in 255 and 163 genes that were down- and up-regulated, respectively (Table 1). GO functional analyses of the down-regulated genes performed at level 2 showed that cell (GO:0005623), membrane (GO:0016020) and organelle GO:0043226) were the GO terms associated with the cellular component category, metabolic process (GO:0008152), single-organism process (GO:0044699) and cellular process (GO:0009987) were associated with the biological process category, and catalytic activity (GO:0003824) and binding (GO:0005488) were associated with the molecular function category (Figure 2). Similarly, the up-regulated genes with these GO functional classifications were also predominant (Table S3).

### 2.4. GO Enrichment and KEGG Metabolic Pathway Analysis of the Differentially Expressed Genes

**Figure 3:**
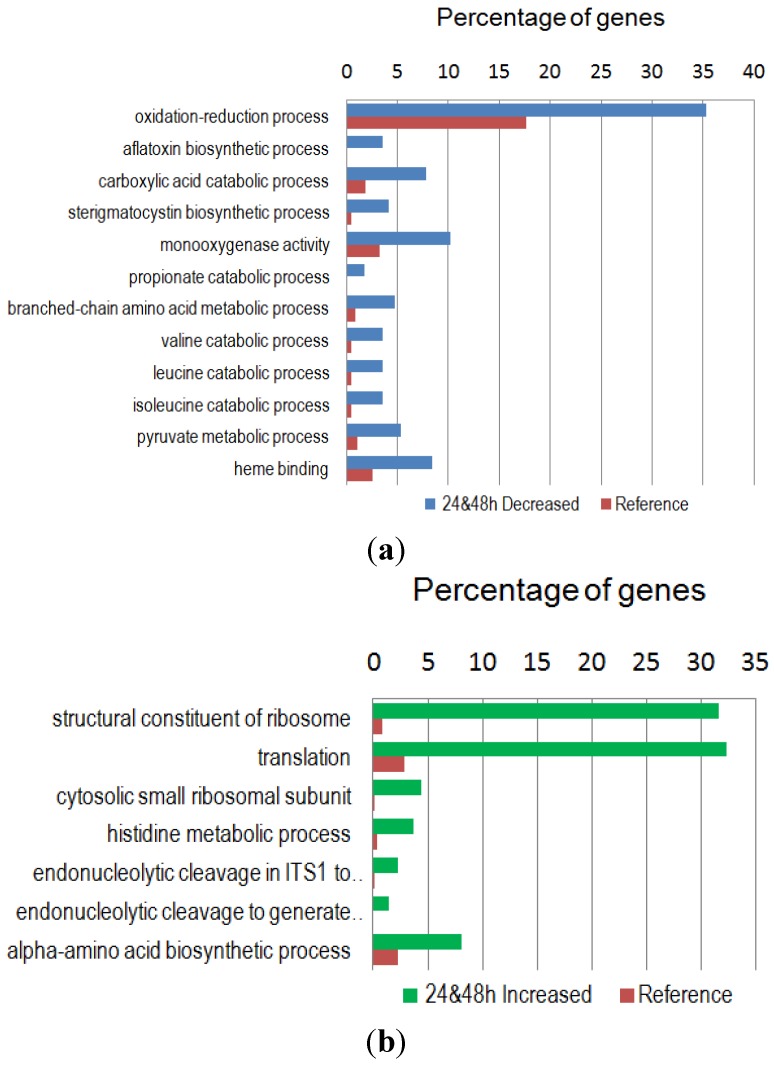
Functional enrichment analyses of differentially expressed genes: (**a**) The 255 significantly down-regulated genes. The propionate catabolic process is the 2-methylcitrate cycle. (**b**) The 163 significantly up-regulated genes. Endonucleolytic cleavage: see Section 2.4 for full description. Fisher’s Exact Test with the FDR corrected *p*-value of <0.05 was used. The reference gene set contained the remaining genes with GO annotations, that is, the whole genes with GO annotations minus the down- or the up-regulated genes with GO annotations.

The specific GO terms enriched in the down-regulated gene group were mainly in the biological process category. These included catabolism of branched-chain amino acids of valine, leucine and isoleucine, and in the biosynthetic pathway of polyketide-derived sterigmatocystin and aflatoxin (Figure 3a). The specific GO terms enriched in the up-regulated gene group (Figure 3b) in the cellar component category were specific for the cytosolic small ribosomal subunit. The molecular function was mainly responsible for the structural constituent of the ribosome, *i.e.*, the integrity of ribosome. In the biological processes category, the GO terms were primarily for the production of mature rRNA, which include endonucleolytic cleavage in ITS1 to separate SSU (small subunit)-rRNA from 5.8S rRNA and LSU (large subunit)-rRNA from tricistronic rRNA transcript (SSU-rRNA, 5.8S rRNA, LSU-rRNA), endonucleolytic cleavage to generate mature 3′-end of SSU-rRNA from (SSU-rRNA, 5.8S rRNA, LSU-rRNA), translation, and biosynthesis (anabolism) of α-amino acids. Tables S4 and S5 further show the summaries of the general (detailed) GO terms of the down- and up-regulated genes including IDs, respective categories, FDRs and *p*-values.

The KEGG pathway analysis confirmed that the amino acid metabolic pathways identified in the down-regulated gene group were consistent with the enriched GO terms in the biological processes category including those involved in metabolism branched-chain amino acids (Table 3). In addition, other pathways involved in the metabolism of amino acids with non-polar (serine and threonine) and polar side chain (tryptophan, phenylalanine and glycine) were identified. However, the metabolic pathways associated with the up-regulated gene group were those for basic (histidine, arginine and proline) and acidic (aspartate and glutamate) amino acids.

**Table 3:** KEGG pathways of differentially expressed genes with GO terms enriched.

KEGG Metabolic Pathway ^a^	#Seq/#Enz	Order of Abundance ^b^
**Down-Regulated Gene Group**		
Pyruvate metabolism	7/8	18
Valine, leucine and isoleucine degradation	7/6	32
Propanoate metabolism	6/5	33
Tryptophan metabolism	5/4	7
Glyoxylate and dicarboxylate metabolism	5/4	28
Phenylalanine metabolism	4/3	8
Fatty acid degradation	4/4	10
Glycine, serine and threonine metabolism	4/3	3
ß-Alanine metabolism	4/4	48
**Up-Regulated Gene Group**		
Histidine metabolism	4/6	53
Arginine and proline metabolism	3/5	5
Pyruvate metabolism	3/2	18
Citrate cycle (TCA cycle)	2/1	42
Alanine, aspartate and glutamate metabolism	2/2	21
Phenylalanine, tyrosine and tryptophan biosynthesis	2/5	44

^a^: 74 and 59 GO-enriched genes in the down- and up-regulated gene groups were used, respectively; ^b^: The ordered rank of gene abundance of all 129 KEGG pathways of *A. flavus* [14].

## 3. Discussion

2-PE at high concentrations (0.3%–0.5%) is lethal to bacteria and fungi [8,15,16,17,18]. At a sublethal level, 2-PE delays spore germination and mycelial growth of *A. flavus*. Previous results indicate that 2-PE (1.0 µL/mL) used in the present study has no discernible adverse effects on fungal growth and does not disrupt membrane integrity [8]. Nonetheless, 2-PE at this concentration inhibits the expression of all genes in the aflatoxin gene cluster (Table 2) and the cyclopiazonic acid gene cluster. The marked decrease in the expression of the majority of aflatoxin genes within the first 24 h growth period correlates well with the two- to three-fold decrease in the pathway regulatory genes of *aflR* and *aflJ*, which are required for transcriptional activation of other aflatoxin biosynthetic genes [19,20]. The smaller decrease in the *aflR* and *aflJ* expression at 48 h and 72 h also correlates with a lesser extent of decrease in other gene expression. Global regulatory genes such as *veA* and *laeA* control the expression of *aflR* and *aflJ* [21,22]. Although the transcriptomic data show a slight decrease (<1.5-fold) in the expression of both of *veA* and *laeA* genes, the decrease is not significant (Table S6). The decrease in all aflatoxin gene expression in the treated *A. flavus* likely results from a broad change in physiology and metabolism (see below).

The GO enrichment analysis of the down-regulated gene group shows that GO terms associated with biosynthesis of toxic secondary metabolites (aflatoxin and sterigmatocystin), and catabolism of carboxylic acid and branched-chain amino acids (valine, leucine and isoleucine) are over-represented. These results are indicative of a metabolic flux being channeled toward primary metabolism for synthesis of *C*- and *N*-containing macromolecules. In the up-regulated gene group GO terms associated with ribosome synthesis, translation and anabolism of α-amino acids are enriched. Taken together, the 2-PE treatment promotes active fungal growth. The KEGG metabolic pathway analyses of the GO-enriched down- and up-regulated gene groups also support this explanation (Table 3). Although the pathway of pyruvate metabolism was identified in both down- and up-regulated gene groups, it suggests that pyruvate enters the mitochondrion and metabolized further for acetyl-CoA production. Acetyl-CoA then either enter the citric acid (TCA) cycle to the processes of energy generation or enter biosynthetic pathways for cell growth. The optimal temperature for aflatoxin biosynthesis is 30 °C. In contrast, a higher temperature such as 37 °C favors fungal growth but suppresses aflatoxin gene expression [23]. Previous KEGG pathway analyses indicate that growth at 30 °C favors degradation of propionate, fatty acid, valine, leucine, isoleucine, tryptophan and phenylalanine [14]. Coincidently, the 2-PE treatment, which decreases the expression of all aflatoxin biosynthesis genes, also results in decreased expression of genes in these metabolic pathways that are active at 30 °C (Table 3). The degradation of isoleucine and valine leads to the production of propionate, which is toxic to cells and inhibits cell growth [24]. Although the methylcitrate cycle is a pathway that can metabolize propionate into pyruvate, which then is used as a energy or carbon source for other metabolism [25], the expression of genes of this pathway is also down-regulated (Figure 3A and Table 3- propanoate metabolism). Noticeably, the expression of genes involved in histidine metabolism, arginine and proline metabolism, alanine, aspartate and glutamate metabolism, and phenylalanine, tyrosine and tryptophan biosynthesis is up-regulated in the 2-PE treated *A. flavus*. The expression of genes in these four pathways is also up-regulated when *A. flavus* is grown at 37 °C [14]. The role of amino acid metabolism in aflatoxin biosynthesis is complex. Specific amino acids used as a sole nitrogen or carbon source have different effects on growth and aflatoxin production. Phenylalanine, tyrosine, tryptophan, proline, and asparagine are readily incorporated into aflatoxin in *A. flavus* [26]. The latter two amino acids also increase aflatoxin production [27]. Asparagine is essential for aflatoxin production in *Aspergillus parasiticus*, and it can be replaced by aspartate or alanine [28]. As a supplement, tryptophan (50 mM) in aflatoxin-conducive medium significantly reduced aflatoxin production in *A. flavus*, but tyrosine at the same concentration significantly increases aflatoxin production [29]. In this study, higher activities in the catabolism of non-polar branched-chain amino acids are correlated with active aflatoxin biosynthesis, while metabolism of other basic and acidic amino acids and biosynthesis (anabolism) of non-polar side-chain tryptophan and phenylalanine are associated mainly with fungal growth (Table 3). These results imply that amino acids play different roles in primary metabolism and secondary metabolism.

The mode of action of 2-PE includes inhibition of synthesis of DNA [13,15,30] and disruption of membrane integrity [31,32]. Most recently Liu *et al.* (2014) have reported that a short 2 h exposure of *Penicillium italicum* to a lethal concentration of 2-PE (1.5 μL/mL) induces subcellular changes in hyphae characteristics by vacuolation, degradation of organelles, and leakage in the outer membrane of mitochondria. In the corresponding transcriptomic analysis, the expression of genes associated with ribosome and amino acid biosynthesis is down-regulated and they conclude that inhibition of the amino acids and protein biosynthesis is the cause of the lethal effect of 2-PE [12]. In this study, we show that a subinhibitory concentration of 2-PE to mycelial growth elicits in *A. flavus* totally opposite changes; it promotes protein synthesis for growth as evidenced by the GO enrichment analysis and the result that expression of about 70% (46/67) of genes encoding known or putative ribosomal proteins is increased significantly in the first 48 h (FDR corrected *p*-value < 0.05, data not shown), but it inhibits secondary metabolism, like the decreased expression of all genes in the aflatoxin and cyplopiazonic acid gene clusters. Studies have shown that plant volatiles of aldehydes, ketones, and alcohols from cotton leaf [33], corn [34], and soybean [35] either are inhibitory to fungal growth or are lethal, and some appear to inhibit aflatoxin production. Although morphological and subcellular changes are usually associated with growth inhibitory effects of these volatiles, the underlying mechanism(s) of how aflatoxin production of *A. flavus* or closely related *A. parasiticus* is decreased or abolished when exposed to sublethal levels of volatile(s) remains unclear. Nonetheless, this study shows that active growth and decreased activities in the metabolism of specific amino acids have a bearing on the decrease in all aflatoxin gene expression in the 2-PE treated *A. flavus*.

In the previous transcriptomic study of *A. flavus* treated by decanal [36], the *con* (*conidiation*) genes were found not to be conidia-specific but are associated with stresses because they are highly expressed in sclerotia, which are hardened hyphal aggregates for survival at unfavorable environments. In addition, Con proteins contain a characteristic stress-response KGG repeat motif. Consistently, *con* transcripts are not detected in growing mycelia in *Neurospora crassa* [37]. The greatly decreased expression in the *con* genes in the 2-PE treated *A. flavus* (Table S6) suggests that 2-PE at this low concentration (1 µL/mL) does not render a stress to the fungal cells. Conidiophorogenesis represents the first phase of *Aspergillus* development after the cessation of vegetative growth. The *brlA* gene which encodes a C_2_H_2_ type transcription factor controls the formation of conidiophore [38]. A delay or reduction in the *brlA* expression has been associated with the halting of programmed conidiation [39,40]. The *brlA* expression in the 2-PE treated *A. flavus* is four- to 10-fold lower than that of controls (Table S6), which suggests that the fungus still is at an active vegetative stage. The initiation of aflatoxin biosynthesis, a secondary metabolism, occurs when active growth slows down [41]. These observations give further support to the proposition that decreased aflatoxin gene expression in *A. flavus* mainly results from the stimulating effect by the subinhibitory concentration of 2-PE on fungal growth.

## 4. Experimental Section

### 4.1. Fungal Strain, Medium and Culture Growth

*Aspergillus flavus* NRRL3357 was maintained on Potato Dextrose Agar (PDA, Becton Dickinson, Franklin Lakes, NJ, USA). A fresh spore suspension was prepared in a 0.05% Tween 80 solution. The NYDB growth medium consisted of nutrient broth 8 g, yeast extract 5 g, and glucose 10 g/L. Aliquots of the spore suspension were inoculated into 20 mL of NYDB in a 125 mL flask to a final concentration of 10^5^/mL. For the treatment set, 2-phenylethanol (2-PE, Sigma-Aldrich, St. Louis, MO, USA) was added to a final concentration of 1 µL/mL. For the control and treatment sets three cultures of each were grown at 28 °C on a rotary shaker at 150 rpm. At 24 h, 48 h and 72 h after inoculation, mycelia were harvested, rinsed with cold DEPC-treated water (0.1% solution), dried by vacuum suction, and ground in a chilled mortar with liquid nitrogen until a fine powder was achieved.

### 4.2. Preparation of Total RNA and Sequencing

Total RNA isolation was carried out using RNeasy Plant Mini Kit (Qiagen, Valencia, CA, USA). The RNA samples were treated with Ambion TURBO DNA-free DNase (Ambion, Austin, TX, USA). The purity and concentrations of RNA were examined by a ND-1000 Spectrophotometer (NanoDrop Technologies, Wilmington, Delaware, DE, USA). RNA quality was assessed with an Agilent 2100 Bioanalyzer (Agilent Technologies, Santa Clara, CA, USA) and all were with RIN (RNA Integrity Number) between 6.9 and 7.8. Samples were stored in a −80 °C freezer until use. Total RNA was processed using an Illumina TruSeq RNA Sample Prep kit, following the manufacturer’s instruction (Illumina, San Diego, CA, USA). After various quality control procedures, individual RNA-Seq libraries were pooled at an equal molar ratio based on their respective sample-specific 6-bp adaptors. Pooled RNA-Seq libraries were then sequenced at 50bp/sequence read using an Illumina HiSeq 2000 sequencer as described previously [42]. Raw single-end sequence reads generated were filtered to remove artificial reads, adapters and low quality reads using the Illumina pipeline to generate fastq files. A total of 18 samples (two groups, three time points, and three biological replicates) were sequenced for this study. The filtered sequence reads were deposited to the NCBI Sequence Read Archive under the accession number of SRP056528 and publically accessible.

### 4.3. Mapping Reads to A. flavus Reference Genome and Normalized Gene Expression Levels

Mapping at 80% identity fraction and 80% length fraction using the RNA-Seq module of CLC Genomic Workbench version 8 was performed [43]. All reads were mapped to gene regions of *A. flavus* NRRL3357 [44] and expression values for every gene in the RPKM (Reads Per Kilobase exon model per Million mapped reads) unit [45] were calculated. These values were normalized for total exon length and the total number of matches in an experiment to allow for cross-sample comparisons.

### 4.4. Statistical Analysis of Digital Gene Expression

The statistical analysis was performed using the Empirical Analysis of DGE (Digital Gene Expression) function of CLC Genomics Workbench, which implements the “Exact Test” for two-group comparisons [46]. This method is similar to Fisher’s Exact Test but takes into account overdispersion caused by biological variability. In other words, the “Exact Test” compares the counts in one set of count samples, *i.e.*, sample replicates, against those in another set of count samples. In comparison, Fisher’s Exact Test compares the counts in one sample against those of another. Total count filter cutoff number was set 5. FDR (False Discovery Rate) corrected-*p* values were calculated from the original *p*-values [47]. FDR is the proportion of false positives among all those positive. In this study, 5% of FDR corrected *p*-values was set to be false-positive (*p* < 0.05).

### 4.5. Gene Ontology, Functional Enrichment, and KEGG Metabolic Pathways

Blast2GO [48] was used to assign GO terms to genes differentially expressed at the first 48 h. Functional enrichment analyses were performed on the down-and up-regulated gene groups, which were compared to the remaining genes of the whole genome using Fisher’s Exact Test with Multiple Test Correction of False Discovery Rate at the threshold of 0.05. Genes associated with the enriched GO terms in the down- and up-regulated gene groups were also analyzed using the reference metabolic pathways of the Kyoto Encyclopedia of Genes and Genomes (KEGG) database [49].

## 5. Conclusions

Our functional genomics study shows that the inhibition of aflatoxin production by the low level of 2-PE results from its effect on promoting active growth of *A. flavus*. Metabolism of different amino acids in primary metabolism and secondary metabolism are associated with *A. flavus* growth, development, and aflatoxin production. Noticeably, aflatoxin production requires a higher activity in the catabolism of branched-chain amino acids. Likely, the end products of this degradation pathway such as acetate and propanoate not only serve as precursors that are channeled into aflatoxin biosynthesis but are also used for energy regeneration. Metabolic flux from primary metabolism can impact the expression of genes of secondary metabolism.

## Supplementary Materials

Supplementary materials can be accessed at: http://www.mdpi.com/2072-6651/7/10/3887/s1.
