# Supplementary material for: Suppression of Aflatoxin Biosynthesis in Aspergillus flavus by 2-Phenylethanol Is Associated with Stimulated Growth and Decreased Degradation of Branched-Chain Amino Acids
Source: Toxins (Basel). 2015 Sep 24;7(10):3887–902. doi: 10.3390/toxins7103887 (PMC4626709; doi:10.3390/toxins7103887)
Supplement: Supplementary file 1 [file toxins-07-03887-s001.pdf]

Supplementary Materials

Table S1. Summary of RNA-Seq reads of the study.

Time	Group	Sample	Total #Sequence
24 h	Control	Rep 1	26,922,227
		Rep 2	24,124,756
		Rep 3	24,124,756
	Treated	Rep 1	35,111,269
		Rep 2	34,556,870
		Rep 3	22,697,534
48 h	Control	Rep 1	15,670,624
		Rep 2	37,179,648
		Rep 3	19,611,339
	Treated	Rep 1	27,924,507
		Rep 2	19,631,078
		Rep 3	22,785,425
72 h	Control	Rep 1	21,232,453
		Rep 2	22,961,786
		Rep 3	19,826,732
	Treated	Rep 1	20,577,004
		Rep 2	23,594,166
		Rep 3	14,823,560

Table S2. Genes differentially expressed in the 2-PE treated *A. flavus* at different growth periods.

Expression [a]	Time					
	**24 h**	**48 h**	**72 h**	**24 h & 48 h**	**48 h & 72 h**	**24 h & 48 h & 72 h**
Decreased	1273	1417	1206	385	559	166
Increased	1607	1166	1103	415	486	175

[a]: The original p-value is <0.05.

Table S3. Gene Ontology classification of genes whose expression was significantly increased during the first 48 h growth period when *A. flavus* was treated with 2-PE.

Category	GO ID	Term	#Sequences
Cellular Component	GO:0005623	cell	85
	GO:0043226	organelle	72
	GO:0032991	macromolecular complex	59
	GO:0005576	extracellular region	9
	GO:0031974	Membrane-enclosed lumen	7
Biological Process	GO:0008152	metabolic process	109
	GO:0009987	cellular process	93
	GO:0071840	cellular component organization or biogenesis	55
	GO:0044699	Single-organism process	28
	GO:0016020	membrane	17
	GO:0050896	response to stimulus	10
	GO:0065007	biological regulation	9
	GO:0032501	multicellular organismal process	2
	GO:0023052	signaling	2
Molecular Function	GO:0003824	catalytic activity	63
	GO:0005488	binding	53
	GO:0005198	structural molecule activity	43
	GO:0051179	localization	11
	GO:0032502	developmental process	2
	GO:0009055	electron carrier activity	2
	GO:0001071	nucleic acid binding transcription factor activity	2

Table S4. Summary of enriched genes whose expression was significantly decreased during the first 48 h growth.

GO ID	GO Term	Category	FDR	*p*-Value	#Test	#Ref.	#notAnnot Test	#notAnnot Ref.
0006082	organic acid metabolic process	P	2.92E-04	6.09E-07	43	1032	124	7788
0006090	pyruvate metabolic process	P	3.26E-02	1.73E-04	9	99	158	8721
0006550	isoleucine catabolic process	P	3.26E-02	1.68E-04	6	39	161	8781
0006552	leucine catabolic process	P	3.26E-02	1.68E-04	6	39	161	8781
0006574	valine catabolic process	P	3.26E-02	1.68E-04	6	39	161	8781
0009081	branched-chain amino acid metabolic process	P	3.05E-02	1.33E-04	8	74	159	8746
0009083	branched-chain amino acid catabolic process	P	3.46E-02	1.90E-04	6	40	161	8780
0009403	toxin biosynthetic process	P	6.12E-05	4.71E-08	11	58	156	8762
0009404	toxin metabolic process	P	6.12E-05	4.71E-08	11	58	156	8762
0016054	organic acid catabolic process	P	7.92E-03	2.56E-05	13	165	154	8655
0019629	propionate catabolic process, 2-methylcitrate cycle	P	1.58E-02	6.13E-05	3	2	164	8818
0019748	secondary metabolic process	P	4.89E-04	1.11E-06	17	212	150	8608
0019752	carboxylic acid metabolic process	P	2.83E-04	4.58E-07	43	1021	124	7799
0032787	monocarboxylic acid metabolic process	P	7.72E-05	7.68E-08	26	402	141	8418
0043385	mycotoxin metabolic process	P	2.77E-03	8.06E-06	6	21	161	8799
0043386	mycotoxin biosynthetic process	P	2.77E-03	8.06E-06	6	21	161	8799
0043436	oxoacid metabolic process	P	2.92E-04	5.79E-07	43	1030	124	7790
0044282	small molecule catabolic process	P	1.94E-02	8.17E-05	13	186	154	8634
0044550	secondary metabolite biosynthetic process	P	2.92E-04	6.14E-07	16	179	151	8641

Table S4. *Cont.*

GO ID	GO Term	Category	FDR	*p*-Value	#Test	#Ref.	#notAnnot Test	#notAnnot Ref
0044710	single-organism metabolic process	P	1.49E-03	3.60E-06	93	3357	74	5463
0044711	single-organism biosynthetic process	P	1.94E-02	7.95E-05	35	935	132	7885
0045122	aflatoxin biosynthetic process	P	7.72E-05	9.99E-08	6	8	161	8812
0045460	sterigmatocystin metabolic process	P	1.42E-02	5.07E-05	7	46	160	8774
0045461	sterigmatocystin biosynthetic process	P	1.42E-02	5.07E-05	7	46	160	8774
0046222	aflatoxin metabolic process	P	7.72E-05	9.99E-08	6	8	161	8812
0046395	carboxylic acid catabolic process	P	7.92E-03	2.56E-05	13	165	154	8655
0055114	Oxidation-reduction process	P	6.12E-05	4.95E-08	59	1554	108	7266
1901376	organic heteropentacyclic compound metabolic process	P	6.12E-05	2.91E-08	11	55	156	8765
1901378	organic heteropentacyclic compound biosynthetic process	P	6.12E-05	2.91E-08	11	55	156	8765
0003824	catalytic activity	F	1.96E-03	5.08E-06	128	5293	39	3527
0004497	monooxygenase activity	F	1.45E-02	5.39E-05	17	290	150	8530
0016491	oxidoreductase activity	F	9.86E-05	1.43E-07	60	1643	107	7177
0020037	heme binding	F	3.26E-02	1.74E-04	14	230	153	8590
0046906	tetrapyrrole binding	F	3.26E-02	1.74E-04	14	230	153	8590

Table S5. Summary of enriched genes whose expression was significantly increased during the first 48 h growth.

GO ID	GO Term	Category	FDR	*p*-Value	#Test	#Ref.	#notAnnot Test	#notAnnot Ref
0005622	intracellular	C	1.33E-09	4.38E-12	83	2824	53	6027
0005623	cell	C	2.67E-09	9.67E-12	85	2982	51	5869
0005737	cytoplasm	C	2.45E-16	6.05E-19	65	1337	71	7514
0005840	ribosome	C	2.50E-35	2.06E-38	47	219	89	8632
0015935	small ribosomal subunit	C	2.34E-16	5.40E-19	13	9	123	8842
0022626	cytosolic ribosome	C	1.87E-06	9.22E-09	6	6	130	8845
0022627	cytosolic small ribosomal subunit	C	1.81E-08	7.44E-11	6	1	130	8850
0030529	ribonucleoprotein complex	C	1.62E-32	1.60E-35	51	332	85	8519
0032991	macromolecular complex	C	4.13E-15	1.09E-17	59	1165	77	7686
0043226	organelle	C	4.20E-10	1.31E-12	72	2158	64	6693
0043228	non-membrane-bounded organelle	C	5.08E-26	8.37E-29	55	575	81	8276
0043229	intracellular organelle	C	4.20E-10	1.31E-12	72	2158	64	6693
0043232	intracellular non-membrane-bounded organelle	C	5.08E-26	8.37E-29	55	575	81	8276
0044391	ribosomal subunit	C	6.63E-17	1.42E-19	16	24	120	8827
0044422	organelle part	C	3.50E-03	2.30E-05	30	869	106	7982
0044424	intracellular part	C	3.71E-09	1.41E-11	79	2650	57	6201
0044444	cytoplasmic part	C	2.05E-19	4.05E-22	61	1011	75	7840
0044446	intracellular organelle part	C	3.50E-03	2.26E-05	30	868	106	7983
0044464	cell part	C	2.67E-09	9.67E-12	85	2982	51	5869
0000447	endonucleolytic cleavage in ITS1 to separate SSU-rRNA from 5.8S rRNA and LSU-rRNA from tricistronic rRNA transcript (SSU-rRNA, 5.8S rRNA, LSU-rRNA)	P	2.27E-02	1.80E-04	3	5	133	8846
0000461	endonucleolytic cleavage to generate mature 3′-end of SSU-rRNA from (SSU-rRNA, 5.8S rRNA, LSU-rRNA)	P	2.82E-02	2.27E-04	2	0	134	8851
0000462	maturation of SSU-rRNA from tricistronic rRNA transcript (SSU-rRNA, 5.8S rRNA, LSU-rRNA)	P	1.66E-03	9.82E-06	4	6	132	8845
0000478	endonucleolytic cleavage involved in rRNA processing	P	2.27E-02	1.80E-04	3	5	133	8846

Table S5. *Cont.*

GO ID	GO Term	Category	FDR	*p*-Value	#Test	#Ref.	#notAnnot Test	#notAnnot Ref.
0000479	endonucleolytic cleavage of tricistronic rRNA transcript (SSU-rRNA, 5.8S rRNA, LSU-rRNA)	P	2.27E-02	1.80E-04	3	5	133	8846
0006364	rRNA processing	P	1.75E-03	1.07E-05	8	63	128	8788
0006412	translation	P	9.29E-30	1.22E-32	44	249	92	8602
0006547	histidine metabolic process	P	1.51E-02	1.07E-04	5	27	131	8824
0009058	biosynthetic process	P	3.85E-08	1.84E-10	71	2330	65	6521
0009059	macromolecule biosynthetic process	P	8.05E-06	4.24E-08	50	1511	86	7340
0010467	gene expression	P	3.00E-08	1.38E-10	53	1411	83	7440
0016072	rRNA metabolic process	P	1.89E-03	1.19E-05	8	64	128	8787
0019538	protein metabolic process	P	2.67E-08	1.19E-10	47	1138	89	7713
0022613	ribonucleoprotein complex biogenesis	P	2.57E-40	1.70E-43	49	190	87	8661
0030490	maturation of SSU-rRNA	P	1.66E-03	9.82E-06	4	6	132	8845
0034645	cellular macromolecule biosynthetic process	P	5.52E-06	2.82E-08	50	1492	86	7359
0042254	ribosome biogenesis	P	3.14E-42	1.03E-45	49	168	87	8683
0042274	ribosomal small subunit biogenesis	P	8.85E-06	4.81E-08	6	9	130	8842
0044085	cellular component biogenesis	P	1.47E-30	1.69E-33	51	369	85	8482
0044238	primary metabolic process	P	5.54E-04	3.10E-06	89	4040	47	4811
0044249	cellular biosynthetic process	P	1.33E-08	5.27E-11	69	2163	67	6688
0044260	cellular macromolecule metabolic process	P	1.99E-02	1.45E-04	58	2451	78	6400
0044267	cellular protein metabolic process	P	1.48E-13	4.13E-16	47	798	89	8053
0052803	Imidazole-containing compound metabolic process	P	1.51E-02	1.07E-04	5	27	131	8824
0071704	organic substance metabolic process	P	5.03E-03	3.40E-05	91	4379	45	4472
0071840	cellular component organization or biogenesis	P	9.32E-21	1.69E-23	55	748	81	8103
0090502	RNA phosphodiester bond hydrolysis, endonucleolytic	P	2.27E-02	1.80E-04	3	5	133	8846
1901576	organic substance biosynthetic process	P	2.27E-08	9.71E-11	70	2245	66	6606
1901607	Alpha-amino acid biosynthetic process	P	4.53E-02	3.73E-04	11	202	125	8649
0003735	structural constituent of ribosome	F	7.26E-46	1.20E-49	43	77	93	8774
0005198	structural molecule activity	F	8.88E-41	4.39E-44	43	112	93	8739

Table S6. Temporal change in the expression level of genes associated with development and cyclopiazonic acid biosynthesis.

Gene Name and Product	Feature ID	24 h Fold Change	FDR *p*-Value	48 h Fold Change	FDR *p*-Value	72h Fold Change	FDR *p*-Value
developmental regulator VeA	AFLA_066460	−1.13	1.00	−1.45	0.02	−1.15	1.00
nucleoside diphosphatase velB	AFLA_081490	−1.02	1.00	−1.02	1.00	1.01	1.00
conserved hypothetical protein VelC	AFLA_025780	1.05	1.00	1.03	1.00	−1.14	1.00
developmental regulator VosA	AFLA_026900	−1.04	1.00	1.15	1.00	−1.54	0.88
extracellular developmental signal biosynthesis protein FluG	AFLA_101920	4.97	0.00	−1.09	1.00	−1.72	0.51
developmental regulator FlbA	AFLA_134030	1.02	1.00	1.64	0.29	2.06	0.01
conserved hypothetical protein FlbB	AFLA_131490	1.03	1.00	1.20	1.00	1.27	0.72
C2H2 conidiation transcription factor FlbC	AFLA_137320	−1.81	0.67	−1.02	0.85	1.03	1.00
conserved hypothetical protein FlbD	AFLA_008170	−3.11	1.00	1.25	1.00	1.15	1.00
conserved hypothetical protein FlbE	AFLA_017380	−1.03	1.00	1.31	0.92	−1.06	1.00
G-protein complex alpha subunit GpaA/FadA	AFLA_018340	1.14	0.15	1.54	0.25	1.28	0.60
C2H2 type conidiation transcription factor BrlA	AFLA_082850	−4.10	1.00	−10.27	0.02	−9.83	0.10
transcription factor AbaA	AFLA_029620	1.30	1.00	1.24	1.00	−1.19	1.00
developmental regulatory protein WetA	AFLA_052030	−1.03	1.00	1.26	1.00	−1.34	1.00
conidiophore development protein HymA	AFLA_079710	−1.06	1.00	1.22	1.00	−1.28	0.95
APSES transcription factor StuA	AFLA_046990	−1.20	0.74	1.09	1.00	1.46	0.35
transcriptional regulator Medusa MedA	AFLA_136410	−1.38	0.16	−1.01	0.84	1.28	0.83
Conidiation-specific family protein	AFLA_044790	−79.92	0.00	−3.97	0.00	−7.65	0.00
conidiation protein Con-6, putative	AFLA_044800	−73.88	0.00	−2.46	0.00	−7.59	0.00
Conidiation-specific protein (Con-10), putative	AFLA_083110	−76.51	0.00	−2.42	0.00	−10.53	0.00
sporulation associated protein	AFLA_021090	−5.77	1.00	−2.71	0.47	−2.84	0.66
HLH DNA binding domain protein, putative SclR	AFLA_040260	−1.38	0.00	−1.12	0.27	2.16	0.24
hypothetical protein SspB	AFLA_070890	#DIV/0!	1.00	1.00	1.00	−2.54	1.00
hypothetical protein C6	AFLA_082140	1.00	1.00	1.00	1.00	1.00	1.00
polyketide synthase, putative PKA27	AFLA_082150	−2.67	1.00	−1.57	1.00	−1.31	1.00
FAD dependent oxidoreductase CpaO	AFLA_139470	−8.52	0.00	−2.60	0.00	−2.25	0.01
dimethylallyl tryptophan synthase CpaD	AFLA_139480	−7.82	0.00	−1.67	0.00	−3.49	0.00
hybrid PKS/NRPS enzyme CpaS	AFLA_139490	−10.83	0.00	−3.33	0.00	−5.87	0.00!
